# Surgical treatment of mild to moderately dilated ascending aorta in bicuspid aortic valve aortopathy: the art of safety and simplicity

**DOI:** 10.1186/s13019-020-1068-7

**Published:** 2020-01-17

**Authors:** Peng Zhu, Pengyu Zhou, Xiao Ling, Bright Eric Ohene, Xiao Ming Bian, Xiaoxiao Jiang

**Affiliations:** 1 Department of Cardiovascular Surgery, First hospital, Dalian Medical University, No. 222, Zhongshan Road, Xigang District, Dalian, Liaoning 125000 People’s Republic of China; 2 Department of Cardiovascular Surgery, NanFang hospital, Southern Medical University, GuangZhou, 515000 People’s Republic of China

**Keywords:** Bicuspid aortic valve, Aortopathy, External wrap

## Abstract

**Background:**

Evaluate the safety and efficacy of our modified technique of the extravascular procedure for treating mild to moderately dilated ascending aorta in patients with bicuspid aortic valve (BAV) aortopathy.

**Methods:**

From January 2015 to December 2018,119 consecutive patients with BAV and ascending aorta dilatation (dimension 40 mm~ 45 mm) were diagnosed in our institution. Among these,49 patients received aggressive aortic valve replace (AVR) + ascending aorta wrapped (wrapped group) while the other 70 patients received AVR + ascending aorta replacement (wheat group). All patients clinical and follow up data were collected for 12 months.

**Results:**

Aortic clamping and cardio-pulmonary bypass times were significantly longer in wheat group than wrap group (*P* < 0.001and 0.021,respectively). The first 24 h drainage in wheat group were much more than wrap group(*P* = 0.04). Ascending aorta diameter、left ventricular end diameter and ejection fraction were statistically different between pre- and post-operation (*p* < 0.001) in both groups, but the heart function and complication were no difference during follow up.

**Conclusions:**

External wrapping of the ascending aorta and wheat procedure have good short-term and long-term results in BAV patients with a mild to moderately dilated ascending aorta. The perioperative period results of external wrapping of the ascending aorta for BAV patients were encouraging.

## Introduction

The recent survey revealed that of all the individuals with BAV, 75% of BAV patients will be presenting aortic valve stenosis and dilation of the supra-coronary aorta, 15% aortic insufficiency and dilation of the proximal aortic root, leaving the fate of the remaining 10% undefined [1, 2]. These enlist the absence or presence of raphes, if yes then numbers were noted, spatial position of cusps or raphes, and functional status of the valve [3]. Aortic valve repair avoids anticoagulation and prosthetic valve-related this could provide many advantages for patients with BAV, but the prosthetics valve replacement remains the most reliable choice of treatment [4].

BAV presents a distinct pattern of ascending aorta dilatation a phenomenon relatively well-recognized among this group of patients to be indirectly proportional to associated valvular lesions. Surgical decision or management strategies were subsequently based on the aortic dilatation classifications [5, 6]. Recommendations for the surgical management of dilatation and aneurysm of the ascending aorta in cases of BAV associated aortopathy are clearly stated in the treatment guidelines but still void of specifics [7, 8]. This method basically involves resection and replacement of the aneurysmal aorta with a vascular graft. Only replacement of dysfunctional aortic valve in patients with BAV may not be able to prevent progressive aortic dilation [9]. Modification to a reparative technique which includes replacing the aortic valve, decreasing the aortic diameter by excision of an oval segment, placing a well-tailored Dacron vascular graft around the ascending aorta, and anchoring it with previously placed sutures driven through the sewing ring of the valve prosthesis through the aortic wall, could done by a simple extravascular wrapping of the ascending aorta with a vascular graft may far useful particularly in geriatric subjects or individuals with high radical operative risks [10, 11]. This prophylactic or corrective technique for mild-to-moderately dilated ascending aorta may be an option, is more responsible on the part of the surgeon than a “wait-and-watch” approach [12]. Therefore, in this article, we aimed to evaluate the feasibility and safety of different intervening measure for BAV patients with mild to the moderately dilated aorta.

## Methods

### Patients

This study was approved by the Institutional Review Board and Ethics committee and the requirement for individual patient consent was waived.

Between January 2015 and December 2018, 119 patients with BAV and dilated ascending aorta underwent the aortic valve replacement concomitantly with ascending aorta surgery, were retrospectively reviewed. A mild to moderately dilated ascending aorta was defined as having an aorta ascendens dimension between 40 mm to 45 mm on the computer tomography. Among these, 49 patients received a extravascular wrapping of the ascending aorta with a vascular graft procedure (wrap group) while the other 70 patients received ascending aorta replacement (wheat operation).

Only BAV patients were diagnosed with mild to moderately aorta ascendens dilation without aortic sinus dilation and had undergone elective aortic valve replacement surgery, were involved in this current study. Following approval from the ethical committee and the electronic database was searched, and relevant information from echocardiographic results, cardiac computed tomography (CT), perioperative recording, surgical notes to outpatient records of all the enrolled subjects were extracted. The data were collected through clinic, a telephone conversation, text messaging and home visits during the following-up.

### The surgical decision

The clinical diagnosis for aortic stenosis (AS) was based on the clinical presentation of the patients and in accordance with standard treatment guidelines, example the 2014 guidelines of the American college of cardiology and American heart association (AHA/ACC) for the treatment of Valvular Heart Diseases (VHD) [13]. Surgical strategies and the management of BAV with aortopathy according to the current clinical guidelines were also applied [13]. Patients are divided into two groups because of different surgeons. All the wrap procedures were performed by a single surgeon.

### The surgical procedure

The Wheat operation: Detailed operative techniques and schematic illustrations of wheat procedure were described in previous article [14]. Only include the supracoronary replacement of the ascending aorta and aortic valve replacement.

AVR + Wrap: Intraoperative measurement of the ascending aorta was taken from the sinotubular junction (STJ) to the right innominate artery. The size, the length and the trimming of the Dacron graft were determined by the parameters below; the maximum diameter (D_max_) of the aneurysmal dilatation, the integrity of the aortic wall, the length of the lesser curvature (L_C_) and the greater curvature (G_C_) and the “post-wrapped” diameter (D_PW)_. The product of the “post-wrapped” diameter (D_PW)_ and pie (π) describes approximately post wrapped circumference (C_PW_) of the ascending aorta and this was marked.

**Fig. 1:**
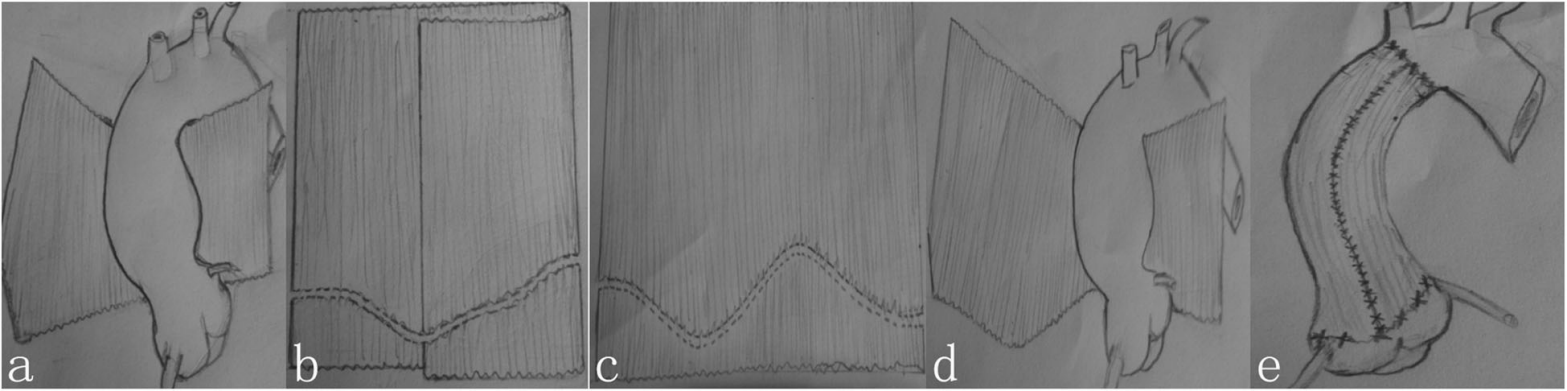
Trimming and wrapping of moderately dilated ascending aorta

For individuals having concomitant cardiac procedure, after completing the procedure, just before wean off CPB, the Dacron graft of predetermined diameter was first to cut longitudinally along the dark marking on side into a spreadsheet, it is transversely placed under the aorta to determine the length of the L_C_ and the G_C_ (Fig. 1a)**,** It was placed beneath the whole length of the ascending aorta, then trimmed based on the length of the L_C_ and G_C_ **(**Fig. 1b, c)**.** The trimmed graft was passed around the ascending aorta covering the entire length from the anterior aortic root to the level of the left coronary artery to the innominate artery (Fig. 1d)**.** The ascending aorta was reduced to the predetermined diameter by approximating the edges of the Dacron with a continuous suture (Fig. 1e). In most cases, wrapping was completed while rewarming. The systolic arterial pressure was kept below 80 mmHg.

### Statistical analysis

For nominal values, data are expressed as a frequency or percentage; Continuous data are given as mean ± standard deviation and range. Ranges are expressed when data were not normally distributed. Differences between groups were compared using unpaired, 2-sided student t-tests for continuous variables. The chi-square test or Fisher’s exact test was used for categoric variables. A *p* value of < 0.05 was defined as statistical significance. Data were analyzed using SPSS software (Version 22.0; SPSS Inc., Chicago, III).

## Results

### Baseline characteristics

Preoperative baseline profiles of the patients were listed in Table 1. Only external wrapping of the ascending aorta was performed in 45.11% (*n* = 49). There was no in-hospital mortality. Most of wrap groups patients age included in this study tended to be relatively young but without statistical difference. Most patients (84.03%) presented with severe aortic stenosis and regurgitation. The incidence of mitral regurgitation between the two groups was the same(*P* = 0.07).

**Table 1:** Sociodemographic characteristics

Characteristic	Total cohort	Wrap(*n* = 49)	Wheat(*n* = 70)	*P* value
Age (years)	60.08 ± 11.7	59.64 + 9.24	61.74+ 10.04	> 0.99
Male	62(52.1%)	25 (51.02%)	37 (52.85%)	0.15
Hypertension	56(47.05%)	23(46.93%)	33(47.14%)	0.63
Diabetes mellitus	29(24.36%)	12(24.48%)	17(24.28%)	> 0.99
Dyslipidaemia	20(16.8%)	5(10.2%)	15(21.43%)	0.045
AR	100(84.03%)	41(83.67%)	59(84.29%)	> 0.99
Mild	65(54.62%)	27(55.10%)	38(54.29%)	> 0.99
Moderate	21(17.64%)	8(16.33%)	13(18.57)	> 0.99
Severe	14(11.76%)	5(10.20%)	9(12.86%)	> 0.99
MR	46(38.65%)	21(42.86%)	26(37.14%)	0.07
Mild	40	19(38.77%)	21(30.00%)	0.32
Moderate	7	2(4.1%%)	5(7.1%)	> 0.99
AD	43.23 ± 5.60	43.11 ± 7.76	43.53 ± 5.76	0.35
AVPG	90.67 ± 23.65	89.87 ± 21.64	92.32 ± 25.63	> 0.99
LVED	54.48 ± 9.87	54.19 ± 10.17	54.74 ± 9.52	0.63
LVEF	49.78 ± 9.37	48.04 ± 9.17	50.97 ± 9.52	0.24
NYHA	2.7 ± 0.9	2.8 ± 0.6	2.7 ± 0.7	0.17

*MR* Mitral regurgitation, *AD* Ascending aorta diameter, *AV* Aortic valve, *PG* Pressure gradient, *LV* left ventricular, *ED* end diastolic diameter, *EF* ejection fraction, *NYHA* New York Heart Association, *AVR* aortic valve replacement

Two techniques for ascending aortic are summarized in Table 2. Aortic cross-clamp (ACC) and cardiopulmonary bypass (CPB) times were longer in TAV group than BAV group (ACC:55.75 ± 9.60 vs. 79.0 ± 5.31 min, *P* < 0.001; CPB:80.18 ± 10.51vs 109 ± 13.38 min, *P* = 0.021). The amount of first 24 h drainages were much more in wheat group than wrap group(512.84 ± 106.36 vs 334.58 ± 78.87 ml, *P* = 0.04).

**Table 2:** Operative characteristics

Characteristics	wrap(n = 49)	wheat(n = 70)	*P* value
ACC time (min)	55.75 ± 9.60	79.0 ± 5.31	< 0.001
CPB time (min)	80.18 ± 10.51	109.77 ± 13.38	0.021
Combined surgery			
MV repair	2(4.1%%)	5(7.1%)	> 0.99
Draining(24 h, ml)	334.58 ± 78.87	512.84 ± 106.36	0.04
Wound complications	2(3.77%)	2(4.54%)	> 0.99
HSAS(d)	8.7 ± 1.2	8.9 ± 1.4	0.63

Data are presented as n(%) or mean ± SD. *MV* mitral valve, *AVR* aortic valve replace, *ACC* aortic cross clamp, *CPB* cardiopulmonary bypass, *HSAO* hospital stay after surgery

### Perioperative results

There was no mortality during perioperative. More than moderate mitral regurgitation was repaired during operation. In patients with Wrap technique (89.07%, *n* = 106), mitral valve repair was used in 2 patients. The first 24 h draining in TAV groups more than BAV groups, but it has not influenced the patients wound recover and out of hospital (Table 2).

The ascending aortic diameter and aortic valve pressure gradient and Left ventricular end-diastolic diameter were obvious decreases in two groups(*P* < 0.001). The left ventricular ejection fraction was increased than the preoperative in two groups(P < 0.001) (Table 3).

**Table 3:** Two groups variable change pre- and post- operation

Variables	wrap(n = 49)		P	wheat(n = 70)		P
	Preoperative	Postoperative		Preoperative	Postoperative	
AD	45.11 + 7.76	34.72 + 4.15	*P* < 0.001	35.53 + 7.76	32.69 + 5.47	*P* < 0.001
AVPG	89.87 + 21.64	44.79 + 8.60	*P* < 0.001	92.32 + 25.63	48.96 + 5.60	*P* < 0.001
LVED	54.19 + 10.17	52.74 + 9.48	*P* < 0.001	54.74 + 9.52	48.83 + 9.07	*P* < 0.001
LVEF	48.04 + 9.17	51.70 + 6.07	*P* < 0.001	50.97 + 9.52	53.91 + 6.49	*P* < 0.001

Data are presented as n(%) or mean ± SD. *AD* ascending aorta diameter, *AVPG* aortic valve pressure gradient, *LV* left ventricular, *ED* end diastolic diameter, *EF* ejection fraction

### Following-up results

The following-up results are listed in Table 4. During the follow-up period, late death occurred in wheat groups(*n* = 1) without a significant inter-group difference. Congestive heart failure occurred in both groups with only one patient, but the difference in the rates was not statistically significant(*P* > 0.99). In terms of warfarin related stroke, there was no difference between the two groups. The ascending aortic diameter without difference between the two groups(P > 0.99).

**Table 4:** Following up results

Variables	Total Cohort	wrap(n = 49)	wheat(n = 70)	P value
NYHA	1.34 ± 0.37	1.31 ± 0.67	1.36 ± 0.44	> 0.99
CHF	2(1.68%)	1(1.88%)	1(1.51%)	> 0.99
AD	33.96 ± 7.84	34.79 ± 4.07	32.67 ± 5.48	> 0.99
Stroke	3	1(1.88%)	2(3.03%)	0.73
Late mortality	1(0.8%)	0	1(1.51%)	> 0.99

Data are presented as n(%) or mean ± SD.AD,Ascending aorta diameter;

*NYHA* New York Heart Association, *CHF* congestive heart failure

## Discussion

Bicuspid aortic valve (BAV) is a congenital variant of the aortic valve morphology whereby there is only two equal or unequal leaflets or cusps aortic valve with a single line of coaptation instead of three [12].

The bicuspid aortic valve is a common congenital cardiovascular malformation affecting 0.5 to 2% of the population [10]. BAV accounts for about 50% of adult cases of aortic stenosis and 70–85% of young adults subjects requiring surgical treatment and frequently aortic regurgitation requiring aortic replacement (AVR) where males are more victims in the ratio of 2:1 [15, 16]. BAV is not just a disorder of heart valve development and limited to abnormal valve morphology but also represents coexistent aspects of a genetic disorder of the aorta and/or cardiac development characterized at an early stage by asymptomatic dilatation of the ascending aorta with or without valvular lesion and later by frequent susceptibility to aneurysm formation of the aorta and to the most dreaded complication, aortic dissection [17].

The clinical presentations in BAV vary from severe valve disease in pediatric patients to asymptomatic valvular or thoracic aortic disease in old age, classical symptoms typically develop in adulthood. Stenosis and Incompetence are the main aortic valve related clinical manifestations, dilated aortopathy (dissection), and acquired complications such as endocarditis [8, 18]. Infantile aortic stenosis is mainly due to a small valvular orifice while that of the pure aortic insufficiency is secondary to a prolapsed leaflet. During adulthood, abnormal shear stress leads to valve calcification and further aortic root dilation in some of the cases. Estimates of late cardiac events include medical and surgical complications were approximately 25% at a mean age of 44 years and 40% at a mean age of 52 years. Cardiac event rates were higher if one or more of the following risk factors were present: age greater than 30 years, moderate or severe aortic stenosis, and moderate or severe aortic incompetence [19]. The folding and creasing of the valves and the turbulent flow contribute to the development of fibrosis and calcification. The combination of these processes results in accelerated disease progression [10].

Surgical intervention depends on the clinical presentation [20, 21]. Valve repair or replacement for valvular lesions, Bentall procedure for root and ascending aortic dilatation and isolated replacement or repair of the ascending aortas. About 27% of adults with BAV and no significant valve disease at baseline required cardiovascular surgery within 20 years of follow-up 22% percent may require intervention within 9 years. Age remains an important determinant of outcomes supporting the notion held by many that eventually, most patients with BAV would require some form of intervention.

Dilatation of the ascending aorta is one of the dreadful clinical complications commonly seen in BAV patients with or without valvular pathology [22]. These aortic dilatation were categorized into 4 patterns: cluster I: aortic root alone; cluster II: tubular ascending aorta alone; cluster III: tubular portion and transverse arch; and, cluster IV: aortic root and the tubular portion with tapering across the transverse arch. Surgical decision or management strategies were subsequently based on each of the above divisions. In BAV disease, the aortic annulus, sinus, and proximal ascending aorta are larger than those found in adults with TAV [14–16]. These differences persist even after adjusting for blood pressure (systolic and diastolic), peak aortic velocities, and left ventricular ejection time.

Valve repair is a new area where as a result of younger age of the insufficiency phenotype subgroups at the time of surgery, raises lots of great concerns, considering this patient group care more about their lifestyle (e.g., sports, exercise, etc.) or even the desire for pregnancy and avoids anticoagulation related risks [23]. Reparation methods such as plication of redundant leaflet tissue, raphe resection, and conjoint cusp reconstruction tricuspidization, pericardial patch augmentation, free-margin reinforcement or resuspension, subcommissural stitching, and suture or ring annuloplasty are considered for this group of patients. The feasibility studies on the midterm durability have been inconsistent. Prosthetic valve replacement for aortic stenosis remains the most reliable surgical resort for BAV valvulopathy in adult patients [24].

Management of BAV and associated complications remain controversial however strategies and the management of BAV with aortopathy according to several current clinical guidelines [25]. The ascending aortic diameter over 4.0 cm in patients with BAV should be considered for concomitant aortic replacement at AVR and dilatation of the ascending aortic diameter of over 0.5 cm per year. The reparative method has become very popular in recent years is the extravascular wrapping of the ascending aorta [11]. It is simple, effective prophylactic or corrective technique though controversial is useful in managing mild-to-moderately dilated ascending patients who are at unacceptably high risk for more complex radical procedures example geriatric or bleeding disorders benefit from reduction ascending plasty. This method principally decreases the aortic diameter by excision of an oval segment, placing a well-tailored Dacron vascular graft around the ascending aorta and anchoring it with previously placed sutures driven through the sewing ring of the valve prosthesis through the aortic wall or just simply doing extravascular wrapping of the ascending aorta far better than the “wait-and-watch” approach. Girding of the external aorta with a synthetic graft or mesh in selected cases of aneurysms can be done, it is favorable because the native epithelial lining is maintained and the integrity of the aortic wall is improved. It is therefore reasonable and lifesaving to offer patients undergoing concomitant cardiac surgery the opportunity to correct any aortic dilatation to prevent adverse future events or reoperation. External wrapping does not prolong the cross-clamping time and reduces blood loss compared to the replacement of the aorta. Size or diameter reduction extravascular wrapping of the ascending aorta in mild to the moderately dilated aorta is a combination of experience and surgical application of fine arts.

The technique itself is easy and does not require long training [26]. It is a convenient and safe procedure that can be used in a selected group of patients whose aortas are not calcified and are not very dilated (> 45 mm). Wrapping technique lower early mortality, compared to the radical replacement (1.51%). Our study result is in agreement with the outcome of the two largest isolated wrapping technique with no early or late aortic related mortality [11].

Biomechanical analysis of external wrapping states that the technique decreases the stress and strain in the aortic wall and may serve a useful purpose in decreasing the risk of aortic dissection [19]. The ascending aortas are pretty normal, at least the endothelial sulface. The incidence of aortic surgical related complications after wrapping procedure is low, the surgical result from our experience registered no dilatation of the mid ascending or enlargement of the sinuses of Valsalva requiring reoperation, no aortic related complication such as dissection, death. Using our trimming technique, wrapping could be extended even few millimeters below the STJ and anchoring the vascular prosthesis proximally and distally will prevent it from dislocating as the few reported complication were mostly associated with the dislocation of the aortic wrap or an aortic root dilatation [9].

Post aortic wrapping revealed that the aortic wall of the moderately dilated ascending aorta (40–45 mm) does not plicate when its diameter is decreased to approximately 30 mm and that the inner surface of the wrapped portion of the aorta simulated as an even surface. It is therefore advisable not to squeeze too much in order to achieve a perfect diameter as this may not do the patient well.

### Study limitation

There are several limitation to this study.(1) The limited follow-up period, the long follow-up time no more than 5 years, so that definite conclusions about the intermediate and long-term outcomes for this wrap procedure are not possible. (2) the patients included in this study were not a homogenous group undergoing isolated AVR and wrap or wheat procedure, but included patients undergoing mitral valve repair. A homogenous group would be preferable from a research aspect, although the rate of mitral valve repair in both groups are similar, and the reported population reflects a real-world experience. (3) A final limitation is the randomized included the patients and surgeons were enrolled in this study, and only one surgeon perform the wrap procedure.all operations are performed by the professional surgeons, and to avoid the influence of surgical experience on the outcome.

## Conclusion

Extravascular wrapping of the aorta is simple, safe and lower surgically related complications, it offers good mid-term and long-term outcome. It may be considered a prophylactic measure for treating BAV patients with mild to moderately dilated ascending aortas or patients at high risk for standard radical aortic replacement procedures.

## Data Availability

The supporting data could immediately become available to the reviewers and editors of the journal.

## Abbreviations

ACC: Aortic cross clamp
AD: Ascending aorta diameter
AS: Aortic stenosis
AV: Aortic valve
AVR: Aortic valve replacement
BAV: Bicuspid aortic valve
BSA: Body surface area
CHF: Congestive heart failure
CPB: Cardiopulmonary bypass
CT: Computed tomography
ED: End diastolic diamenter
EF: Ejection fraction
HSAO: Hospital stay after surgery
LV: Left ventricular
MR: Mitral regurgitation
MV: Mitral valve
NYHA: New York Heart Association
PG: Pressure gradient
VHD: Valvular heart disease
